# Preoperative Measurement of Breast Cancer Overestimates Tumor Size Compared to Pathological Measurement

**DOI:** 10.1371/journal.pone.0086676

**Published:** 2014-01-29

**Authors:** Yi-Zhou Jiang, Chen Xia, Wen-Ting Peng, Ke-Da Yu, Zhi-Gang Zhuang, Zhi-Ming Shao

**Affiliations:** 1 Department of Breast Surgery, Cancer Center and Cancer Institute, Shanghai Medical College, Fudan University, Shanghai, People’s Republic of China; 2 Department of Breast Surgery, Shanghai First Maternity and Infant Hospital, Tongji University School of Medicine, Shanghai, People’s Republic of China; University of Texas MD Anderson Cancer Center, United States of America

## Abstract

**Background:**

Tumor size is one of the most important factors in making clinical and pathological assessment of breast cancer. In the present study, we aimed to determine whether the preoperative measurement of tumor size, by imaging modalities, deviate from the postoperative pathological measurement in breast cancer.

**Patients and Methods:**

1296 patients diagnosed with invasive ductal breast carcinoma (IDC) during 2007 and 2009 were involved. Pre- and postoperative measurements of tumor size were compared using paired t-test and Chi-square test.

**Results:**

The mean maximum diameters of tumors by imaging modalities and pathology were 27.9 mm and 22.4 mm, respectively. There was a statistically significant difference of 5.5 mm (95% CI: 4.7–6.2, p<0.001) between them. The discordance between pre- and post-surgical measurements of tumor size had significant effect on choosing surgery type, causing less application of breast conserving therapy (p<0.0001).

**Conclusion:**

Compared to pathological size, preoperative measurement by imaging modalities tends to overestimate tumor size. These differences could have implications in the treatment of patients with breast cancer.

## Introduction

Tumor size is one of the most important factors in making clinical and pathological assessment of breast cancer. The majority of NCCN staging system (TNM) focused on the T status [1]. Tumor size may influence patients’ T status, thus having an impact on subsequent surgical and oncological management [2]. Therefore, the accuracy of pre-surgical measurement of tumor size in breast cancer becomes crucial.

Among all the existing pre-surgical imaging modalities for breast cancer, magnetic resonance imaging (MRI) is considered to be more accurate than ultrasound and mammography. Pathological measurement, however, is regarded as the gold standard of tumor size measurement.

To our knowledge, there have already been a few researches examining the concordance between pre- and post-surgical tumor size measurements in breast cancer [3]–[5] or some other cancers [6], [7]. Conclusions are that there is a statistically significant difference between imaging and pathological measurement. However, most of the existing researches are lack of enough sample size (less than 200 patients); here, we designed this study with a larger sample size of 1296 patients, aiming to draw a more convincing conclusion.

## Materials and Methods

### Patients

A retrospective study was performed on 1296 female breast cancer patients with histologically confirmed invasive ductal breast carcinoma (IDC) from Fudan University Shanghai Cancer Center (FUSCC) between January 1, 2007 and December 31, 2009. Patients had operable tumors measured by MRI, ultrasound or mammography were enrolled, and those with inoperable lesions were excluded. All the enrolled patients underwent surgical treatment at FUSCC. Surgical resection of the tumor was performed two days after the imaging measurement. Follow-up was completed on September 1, 2013. The median length of follow-up was 44 months (range from 2 to 75 months). Our definition of disease-free survival (DFS) events included: the first recurrence of disease at a local, regional, or distant site; the diagnosis of contralateral breast cancer; and death from any causes. Overall survival (OS) was calculated from the date of diagnosis to the date of death or last follow-up. Patients without events or death were censored at the last follow-up. One hundred and eighty patients had DFS events and 134 had died at the end of follow-up. Molecular subtypes of breast cancer according to immunohistochemical profile were categorized as follow: luminal A = (ER or PR) +, HER2− and Ki67<14%; luminal B = (ER or PR)+and (HER2+ or Ki67≥14%); HER2+ = ER-, PR- and HER2+; and basal-like = ER-, PR-, HER2− and (CK5/6+ or EGFR+) [8], [9]. Staining and interpretation of ER, PR, HER2, Ki-67, EGFR, and CK5/6 have been previously described [10]. Our study was approved by the independent ethical committee/institutional review board of FUSCC (Shanghai Cancer Center Ethical Committee). All patients gave their written informed consent before inclusion in this study.

### Preoperative Tumor Size Measurement

As MRI was reported the most accurate method to measure tumor size before surgical treatment, patient’s largest diameter dimension from MRI report was utilized as the pre-surgical size if available. For those without MRI report, largest diameter from ultrasound or mammography reports was chosen in descending priority. The MRI scans and mammography reports were interpreted by an experienced breast radiologist (YJG). Tumor contours were delineated using a semi-automated segmentation algorithm and adjusted based on the radiologist’s input. Based on the tumor perimeter, maximal tumor linear diameter was calculated automatically. The largest diameters of tumors from ultrasound were measured independently by two experienced physicians (CC and LHX), and any discrepancy were adjudicated by ZMS.

### Pathological Specimens and Size Measurement

Pathological data were obtained from the pathological report of gross specimen. All specimens were received fresh and converted to sections within one hour following resection. The largest diameter dimension of the resected breast tumor was utilized and measured by a physician (WTY) or a physician’s assistant (BHY) with a standard ruler.

### Statistical Analysis

Paired Student’s t-test was performed to examine the difference of measured sizes between imaging modalities and pathological report. Mean, median and ranges for both methods were obtained. Bland-Altman plot and linear regression analysis were used to explore the agreement between the two measurements. Chi-square test was applied to determine whether there was a significant interaction between the measuring discrepancy and certain clinicalpathological factors, or to see whether the measuring discrepancy influenced surgery type. Survival curves were constructed using the Kaplan-Meier method, and the univariate survival difference was determined by the log-rank test. All statistical analysis was performed using Stata statistical software, version 10.0 (StataCorp, College Station, TX, USA). A two-sided p<0.05 was considered statistically significant.

## Results

### Differences between Pre-surgical and Pathological Tumor Sizes

**Figure 1:**
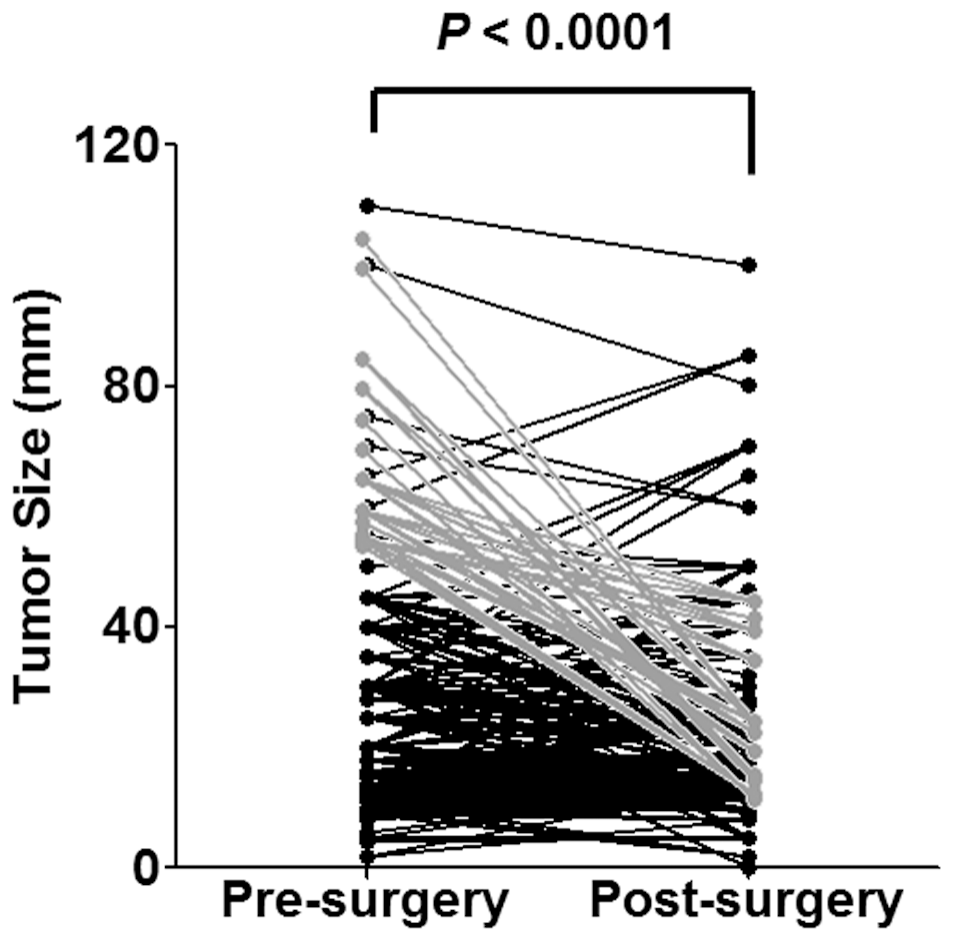
Paired t-test of pre- and post-surgical tumor sizes in 1296 breast cancer patients. Gray dots and lines indicated 92 patients with preoperative diameters >50 mm and postoperative <50 mm.

Of all the 1296 patients enrolled in our study, the proportions of MRI, ultrasound and mammography adoption were 55.9% (724 cases), 24.7% (320 cases) and 19.4% (252 cases), respectively. The mean diameter of pre-surgical measurement was 27.9 mm (range from 2.0 to 110.0 mm) while that of the pathological measurement was 22.4 mm (range of 0.0 to 100.0 mm) (**Table 1**). Paired t-test was carried out to compare pre- and post-surgical measurement, and we found a statistically significant difference of 5.5 mm (95% CI: 4.7–6.2, p<0.0001) (**Figure 1**). The relative percent difference ((pre-surgical – post-surgical)/pre-surgical*100%) was 19.6% (**Table 1**).

**Table 1:** Measurements of pre- and post-surgical tumor sizes of 1296 breast cancer patients.

Measurement	Mean	SD	Median	Min	Max
Pre-surgery, mm	27.9	16.17	25.0	2.0	110.0
Post-surgery, mm	22.4	13.52	18.0	0.0	100.0
Difference, mm	5.5	14.2	7.0	−40.0	70.0
Relative difference (%)	19.6	29.4	15.4	−42.8	81.6

*SD* Standard Deviation.

**Figure 2:**
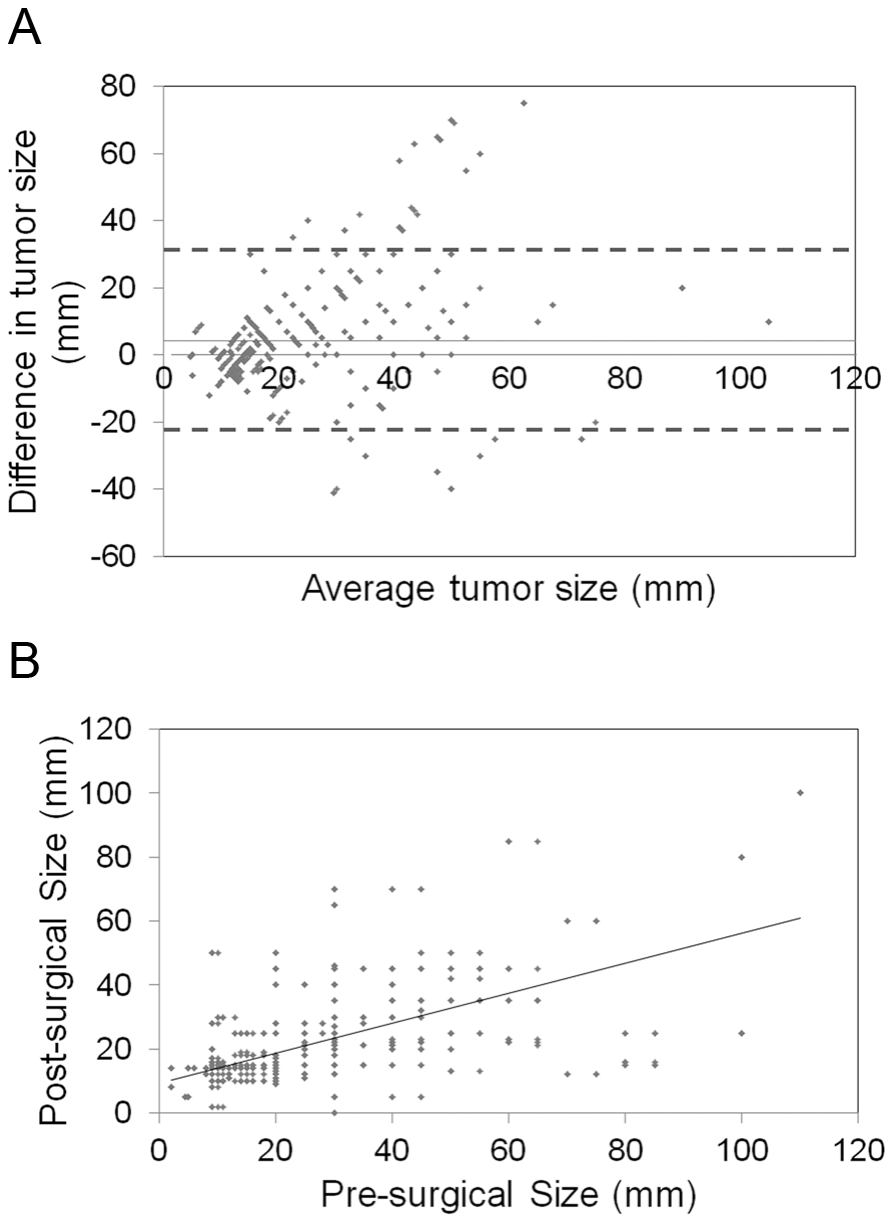
The association between pre-surgical and pathological tumor sizes in 1296 breast cancer patients. A. Bland-Altman plot of difference and average of pre- and post-surgical tumor sizes. B. Scatter plot and linear regression of the observed pre-surgical tumor size (x axis) and pathological measurement (y axis).

Bland-Altman plot was used to measure the difference of pre- and post-surgical tumor sizes against the mean value of them (representing estimated true tumor size) (**Figure 2A**). The mean difference between the two measurements was shown with a solid line at 5.5 mm; the lower and upper 95% limits of agreement were shown by the dashed lines at −21.4 mm and 31.3 mm, respectively. However, the magnitude of the difference did not seem to differ by average value of them since there was no apparent pattern on the plot.

Furthermore, linear regression analysis was performed to further characterize the correlation between the pre- and post-surgical sizes of breast tumor. The results suggested a significant relationship between pre- and post-surgical measurement (r = 0.72, 95% CI: 0.59–0.83, p<0.0001) (**Figure 2B**).

### Association between Clinicopathological Characteristics and Tumor Size Discordance

Of the preoperative tumor sizes of the total 1296 patients, 728 (56.2%) had been overestimated and 348 (26.8%) had been underestimated both by more than 5 mm in imaging measurements, the rest 220 (17.0%) had difference within 5 mm. Accordingly, we divided all patients into three groups. **Table 2** showed clinicopathological characteristics (age, pathological tumor size, grade, lymph node status, molecular subtype and surgery type) that we considered might be responsible for the discrepancy of pre- and post-surgical measurements of tumor size. However, Chi-square test showed that none of the listed factors was significantly correlated with the discordance of tumor size measurements. Since MRI, ultrasound, and mammography do not have the same degree of detail or accuracy, we further questioned if the misjudgment in preoperative size was different in the different imaging modalities (**Table 2**). Chi-square test revealed no significant association between different imaging modalities and the misjudgment (p = 0.111).

**Table 2:** Association between clinicopathological characteristics and tumor size discordance.

	Pre <Post	Concordant	Pre >Post	Total	p-value
Characteristics	by >5 mm	by ±5 mm	by >5 mm	n = 1296	
	n = 348 (%)	n = 220 (%)	n = 728 (%)		
Age, y					
<50	154 (27.7)	90 (16.2)	312 (56.1)	556	0.735
≥50	194 (26.2)	130 (17.6)	416 (56.2)	740	
Pathological size, mm					
≤20	120 (28.0)	84 (19.6)	288 (67.4)	428	0.254
>20 to ≤50	196 (26.9)	112 (15.4)	356 (48.9)	728	
>50	32 (22.9)	24 (17.1)	84 (60.0)	140	
Grade					
I	60 (32.6)	36 (19.6)	88 (47.8)	184	
II	118 (26.3)	72 (16.1)	258 (57.6)	448	0.452
III and UD	156 (26.7)	100 (17.1)	328 (56.3)	584	
Unknown	24 (6.9)	12 (5.4)	44 (6.0)	80	
LN status					
Negative	220 (63.2)	126 (57.3)	464 (63.7)	760	0.501
Positive	104 (29.9)	74 (33.6)	212 (29.1)	440	
Unknown	24 (25.0)	20 (20.8)	52 (54.2)	96	
Molecular subtype					
Luminal A	124 (27.2)	84 (18.4)	248 (54.4)	456	
Luminal B	76 (26.4)	44 (15.3)	168 (58.3)	288	0.985
ERBB2+	56 (25.9)	36 (16.7)	124 (57.4)	216	
Basal-like	64 (27.1)	40 (16.9)	132 (56.0)	236	
Unknown	28 (28.0)	16 (16.0)	56 (56.0)	100	
Imaging modalities					
MRI	182 (25.1)	140 (19.3)	402 (55.6)	724	0.111
Ultrasound	91 (28.4)	44 (13.8)	185 (57.8)	320	
Mammography	75 (29.8)	36 (14.3)	141 (55.9)	252	
Surgery type					
None	8 (28.6)	4 (14.3)	16 (57.1)	28	0.102
Lumpectomy	144 (31.3)	76 (16.5)	240 (52.2)	460	
Mastectomy	196 (24.3)	140 (17.3)	472 (58.4)	808	

*LN* lymph node, *UD* undifferentiated.

### Effect of the Discordance of Size Measurement on Surgery Type

**Figure 3:**
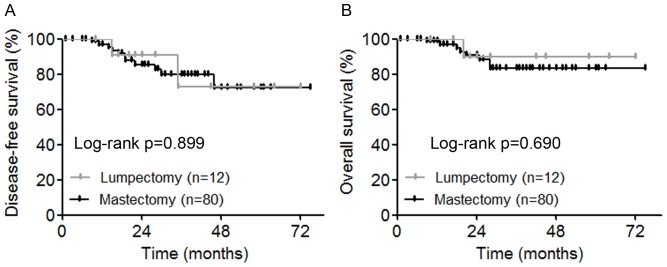
Kaplan-Meier estimates of disease-free survival (A) and overall survival (B) between the 12 patients received lumpectomy and the 80 patients received mastectomy.

Since the latest edition of NCCN clinical practice guidelines of breast cancer listed tumor over 5 cm as one of the relative contraindications for breast conserving therapy (BCT) [2], we selected those patients with tumors measured >5 cm by imaging modalities and <5 cm by pathological measurement. Chi-square test showed that for these 92 (7.1%) patients, the proportion of lumpectomy (12 of 92, 13.0%) was significantly lower than that of those with pre- and post-surgical sizes both less than 5 cm (440 of 1156, 38.1%, p<0.0001) (**Table 3**). All the other factors listed in **Table 3** (age, grade, lymph node status and molecular subtype) had no significant difference between the two groups. Furthermore, in the 92 patients with tumors measured >5 cm by imaging modalities and <5 cm by pathological measurement, we performed Kaplan-Meier plot to examine whether there was any difference of prognosis between the 12 patients receiving lumpectomy and the 80 patients receiving mastectomy. Kaplan-Meier estimates revealed no statistical difference in either DFS (p = 0.899) or OS (p = 0.690) between the two groups (**Figure 3**). Due to the relatively small sample size, we did not calculate adjusted hazard ratios with 95% confidence intervals using Cox proportional hazards models. Taken together, the overestimation of tumor size was correlated with less application of BCT.

**Table 3:** Effect of the discordance of size measurement on surgery type.

Characteristics	pre >50 mm	pre <50 mm	p-value
	post <50 mm	post <50 mm	
	n = 92 (%)	n = 1156 (%)	
Age, y			
<50	36 (39.1)	520 (45.0)	0.277
≥50	56 (60.9)	636 (55.0)	
Grade			
I	16 (17.4)	160 (13.8)	
II	32 (34.8)	432 (37.4)	0.703
III and UD	40 (43.5)	528 (45.7)	
Unknown	4 (4.3)	36 (3.1)	
LN status			
Negative	60 (65.2)	712 (61.6)	0.559
Positive	28 (30.4)	408 (35.3)	
Unknown	4 (4.4)	36 (3.1)	
Molecular subtype			
Luminal A	36 (39.1)	436 (37.7)	
Luminal B	20 (21.8)	264 (22.8)	0.846
ERBB2+	12 (13.0)	196 (17.0)	
Basal-like	20 (21.8)	220 (19.0)	
Unknown	4 (4.3)	40 (3.5)	
Surgery type			
None	0 (0)	24 (2.1)	**<0.0001**
Lumpectomy	12 (13.0)	440 (38.1)	
Mastectomy	80 (87.0)	692 (59.8)-	

*LN* lymph node, *UD* undifferentiated.

## Discussion

Our study demonstrated that preoperative measurements tended to overestimate the actual tumor size in breast cancer patients. None of the clinicopathological characteristics was significantly correlated with the discordance of tumor size measurement. The overestimation of tumor size resulted in less application of BCT.

The reason for overestimation of tumor size was complex. Blood and fluid drained from a specimen once it was removed from the body, which might result in shrinkage of the specimen. Furthermore, infiltration and/or edema around the tumor might be measured preoperatively causing overestimation of the maximum dimension.

Previous study showed that MRI tumor size measurement matched with pathological size in more than half of patients, especially in patients with tumors less than 2 cm [3]. There was also studies showing that MRI overestimating tumor size in low grade breast cancer [4]. However, our data hints that in all three groups (namely underestimating, concordant and overestimating), the constituent ratios of age, pathological tumor size, grade, lymph node status, molecular subtype and surgery type were nearly equal. The reason why our result differed from previous studies might be attributable to the large sample size.

As mentioned above, tumor size is one of the most important factors in making assessment and oncological management of breast cancer including choosing surgery type. BCT is now becoming one of the standard breast cancer treating methods. Women receiving BCT were reported more satisfied with their body image and with better physical and role function than those receiving mastectomy [11], [12]. Those who were young and highly educated benefited even more from BCT [13]. The latest edition of NCCN clinical practice guidelines of breast cancer listed tumor over 5 cm of its diameter as one of the relative contraindications for BCT [2]. Thus, the accuracy of preoperative tumor measurement is of great importance in deciding patients’ eligibility for conserving their breasts. In the present study, we focused on the effect of measuring discrepancy on BCT application. We noticed that in patients whose breast tumor been overestimated to >5 cm by imaging modalities, BCT was significantly less performed than in those whose preoperative tumor size was less than 5 cm.

One of the limitations of the current study is that there might be some potential factors we didn’t take into consideration in our statistical analysis. Furthermore, since personal situation differs a lot from one to another, it is hard to build a precise mathematic mode to assess tumor size accurately and noninvasively. If possible, more comprehensive sample data should be accessed and systematic statistical analysis could be applied to build a predictive mode.

Our study proved the discrepancy of imaging and pathological measurements of tumor size with large sample size in breast cancer patients. Furthermore, the application of BCT might be decreased by the inaccuracy of imaging modalities. These differences could have implications in the treatment of patients with breast cancer.
